# Cardiovascular Vulnerability, Including Heart Failure Risk, in Breast Cancer Surgery: The Role of Operative Technique, Frailty, and Postoperative Complications

**DOI:** 10.3390/medicina62050877

**Published:** 2026-05-03

**Authors:** Andrei Marginean, Madalin Margan, Dragos-Mihai Gavrilescu, Diana-Maria Mateescu, Ioana Cotet, Cristina Tudoran, Dan Alexandru Surducan, Camelia-Oana Muresan

**Affiliations:** 1 Department of Surgery, “Dr. Victor Popescu” Emergency Military Hospital, 9 Gheorghe Lazăr Street, 300080 Timișoara, Romania; 2 Discipline of Public Health, Department of Functional Sciences, Victor Babes University of Medicine and Pharmacy, 300041 Timisoara, Romania; margan.madalin@umft.ro; 3 Department of Orthodontics, Dental District, Strada Zăgazului Nr. 3, One Floreasca Vista, Sector 1, 014261 Bucharest, Romania; 4 Doctoral School, Department of General Medicine, “Victor Babes” University of Medicine and Pharmacy, Eftimie Murgu Square 2, 300041 Timisoara, Romaniaioana.cotet@umft.ro (I.C.); 5 Center of Molecular Research in Nephrology and Vascular Disease, Faculty of the of Medicine, “Victor Babes” University of Medicine and Pharmacy, E. Murgu Square, Nr. 2, 300041 Timisoara, Romania; 6 Department VII, Internal Medicine II, Discipline of Cardiology, University of Medicine and Pharmacy “Victor Babes” Timisoara, E. Murgu Square, Nr. 2, 300041 Timisoara, Romania; 7 County Emergency Hospital “Pius Brinzeu”, L. Rebreanu, Nr. 156, 300723 Timisoara, Romania; 8 Department of Neuroscience, Forensic Medicine, Bioethics, Deontology and Medical Law, “Victor Babes” University of Medicine and Pharmacy, 300041 Timisoara, Romania; 9 Institute of Legal Medicine, 300041 Timisoara, Romania; 10 Ethics and Human Identification Research Center, Department of Neurosciences, “Victor Babes” University of Medicine and Pharmacy, 300041 Timisoara, Romania

**Keywords:** breast cancer surgery, heart failure, cardiovascular risk, frailty, perioperative complications, breast reconstruction, cardio-oncology, surgical risk stratification

## Abstract

*Background and Objectives*: Breast cancer surgery is increasingly performed in older patients with multimorbidity, in whom cardiovascular disease and frailty may substantially modify perioperative risk, including vulnerability to heart failure decompensation and other major medical complications. However, most available studies report global perioperative complication rates and composite medical endpoints, with heart failure events only rarely captured as dedicated outcomes, and operative technique, cardiovascular comorbidity, and frailty are often treated as separate domains rather than components of an integrated risk framework. *Materials and Methods*: We conducted a systematized narrative review with a structured literature search in PubMed/MEDLINE, Scopus, and Web of Science from inception to 31 January 2026, including original studies of adult patients undergoing breast-conserving surgery, mastectomy, and/or reconstruction that reported early postoperative outcomes in relation to comorbidities, cardiovascular risk, or frailty. Eligibility assessment, data extraction, and qualitative synthesis followed key PRISMA 2020 principles, and findings were organized into three prespecified domains: surgical complexity, cardiovascular vulnerability (including patients with heart failure where reported), and frailty. *Results*: Nineteen studies (retrospective cohorts, registry-based analyses, and large database studies, primarily ACS NSQIP) met inclusion criteria, encompassing diverse breast surgery populations, including elderly, metastatic, and reconstructive cohorts. Across datasets, escalation from breast-conserving surgery to mastectomy and then to increasingly complex reconstruction was associated with a stepwise increase in perioperative complications, reoperations, bleeding, and, in selected series, catastrophic events. Preexisting cardiovascular disease and systemic vascular pathology significantly amplified postoperative morbidity even in procedures considered low or intermediate cardiac risk, with signals that patients with underlying heart failure carry particularly heightened vulnerability, although HF-specific events were infrequently reported as separate endpoints. Frailty, mainly assessed using modified frailty indices, consistently emerged as a strong, age-independent predictor of 30-day complications, mortality, and readmissions across surgical types, including both breast-conserving and reconstructive procedures. *Conclusions*: Early postoperative outcomes after breast cancer surgery are associated with the interaction between surgical complexity, cardiovascular comorbidity (with limited HF-specific reporting), and frailty rather than by operative technique alone. In this context, our synthesis primarily reflects overall cardiovascular vulnerability in comorbid and frail patients, with heart failure risk inferred indirectly from the available data. These findings support a patient-centered, risk-adapted surgical strategy in which the extent and timing of surgery and reconstruction are tailored to each patient’s cardiovascular profile and frailty status, with preferential use of breast-conserving or less complex procedures in vulnerable individuals. Integrating standardized frailty assessment and cardio-oncologic evaluation into preoperative workflows, and prospectively validating this tri-axial framework in dedicated cohorts, may improve perioperative risk stratification and reduce the burden of postoperative medical complications in an aging breast cancer population.

## 1. Introduction

Breast cancer remains the most frequently diagnosed malignancy among women worldwide and a leading cause of cancer-related morbidity and mortality, with over 2.3 million new cases annually [1,2]. Advances in early detection and multimodal therapy have significantly improved survival, resulting in a growing population of patients undergoing surgical treatment with increasingly complex clinical profiles. Surgical management—ranging from breast-conserving surgery (BCS) to mastectomy with or without reconstruction—continues to represent a cornerstone of curative-intent therapy, with long-term randomized trials demonstrating comparable oncologic outcomes between conservative and radical approaches in appropriately selected patients [3,4].

In parallel with improved oncologic outcomes, the cardiovascular health of patients with breast cancer has emerged as a major determinant of both short- and long-term prognosis. Cardiovascular disease (CVD) is highly prevalent in this population, particularly among older individuals, and shares multiple risk factors with breast cancer, including age, obesity, and metabolic dysfunction [5]. In addition, exposure to cardiotoxic oncologic therapies—such as anthracyclines, HER2-targeted agents, and thoracic radiotherapy—further increases cardiovascular vulnerability, contributing to the growing field of cardio-oncology [6,7].

From a perioperative perspective, cardiovascular comorbidity represents a key determinant of surgical risk. Even in non-cardiac procedures traditionally considered low to intermediate risk, such as breast surgery, underlying cardiovascular disease has been associated with increased rates of postoperative complications and mortality [8]. Established risk prediction models, including the Revised Cardiac Risk Index, as well as contemporary guideline-based approaches, consistently identify heart failure, ischemic heart disease, and reduced functional capacity as major predictors of adverse perioperative outcomes [9,10].

Beyond patient-related factors, the extent and complexity of surgical intervention also influence perioperative risk. While breast-conserving surgery is generally associated with lower morbidity, more extensive procedures—such as mastectomy, particularly when combined with immediate reconstruction—are associated with longer operative times and greater physiological stress, potentially increasing the risk of systemic complications [3,4]. The ongoing evolution of oncoplastic and reconstructive techniques has improved aesthetic and oncologic outcomes; however, these advances necessitate careful risk stratification, especially in patients with significant comorbid burden.

Frailty has emerged as a critical concept in surgical oncology, reflecting reduced physiological reserve and increased susceptibility to stressors such as surgery. Independent of chronological age, frailty has been consistently associated with adverse postoperative outcomes, including complications, prolonged hospitalization, and mortality [11,12]. Importantly, frailty frequently coexists with cardiovascular disease, creating a cumulative effect that amplifies perioperative vulnerability. Commonly used assessment tools include the modified frailty index (mFI), the Fried phenotype, and the Clinical Frailty Scale, each capturing different dimensions of physiological reserve.

Despite these advances, a significant gap remains in the current literature. Most studies evaluating outcomes after breast cancer surgery focus primarily on surgical complications or composite endpoints, with limited attention to specific cardiovascular outcomes such as acute heart failure decompensation or major adverse cardiac events. Furthermore, the interaction between surgical technique, cardiovascular comorbidity burden, and frailty has not been comprehensively evaluated in an integrated manner.

Given these limitations, a comprehensive synthesis of the available evidence is warranted. A better understanding of how operative strategy interacts with cardiovascular risk and patient frailty is essential for optimizing perioperative management, improving risk stratification, and guiding individualized clinical decision-making.

To our knowledge, this is the first review to integrate surgical complexity, cardiovascular vulnerability, and frailty into a unified, clinically applicable perioperative risk framework specifically in the context of breast cancer surgery. Therefore, the aim of this study is to synthesize available evidence on the interaction between operative technique complexity, cardiovascular comorbidity (including heart failure risk), and frailty, and to develop an integrated, patient-centered framework for perioperative risk stratification in breast cancer surgery.

## 2. Materials and Methods

### 2.1. Study Design

This study was designed as a narrative review with a structured, systematized search rather than a formal systematic review with meta-analysis. The research question was intentionally broad and integrative, aiming to explore the complex interplay between surgical technique complexity, cardiovascular vulnerability (including heart failure), and frailty across heterogeneous populations and study designs. The available evidence consists predominantly of retrospective cohorts, registry-based analyses, and large administrative database studies, characterized by substantial clinical, methodological, and outcome heterogeneity (including differences in frailty indices, variability in cardiovascular reporting, and non-uniform definitions of postoperative complications), which would render quantitative pooling and meta-analysis inappropriate or potentially misleading. The primary objective of this review was therefore to develop a clinically actionable, integrated conceptual framework rather than to provide a narrowly focused quantitative synthesis of effect sizes.

This hybrid “systematized narrative review” approach, which applies systematic methods to literature identification, screening, and data extraction while allowing qualitative synthesis and integrative interpretation, follows recommendations for complex, multidisciplinary topics where formal meta-analysis is inappropriate due to heterogeneity. To maximize transparency and reproducibility, we applied selected key principles from the PRISMA 2020 framework—specifically regarding search strategy, study selection, and flow diagram reporting—recognizing that the full PRISMA checklist is designed for formal systematic reviews and was therefore not applied in its entirety.

### 2.2. Literature Search Strategy

A comprehensive literature search was performed in PubMed/MEDLINE, Scopus, and Web of Science from database inception to 31 January 2026. The search was last updated on 31 January 2026. The search strategy combined Medical Subject Headings (MeSH) terms and free-text keywords related to breast cancer surgery, cardiovascular disease, heart failure, perioperative risk, postoperative complications, and frailty. No study design filters were applied at the search stage in order to maximize sensitivity.

The core search strategy used for PubMed/MEDLINE was as follows:

(“breast cancer surgery” OR mastectomy OR “breast-conserving surgery” OR “oncoplastic surgery” OR “breast reconstruction”) AND (“cardiovascular disease” OR “heart failure” OR “cardiac risk” OR “perioperative risk” OR “postoperative complications” OR frailty OR “frailty index”)

Equivalent search strategies, adapted to database-specific syntax, were applied in Scopus and Web of Science. Searches were limited to English-language publications and human subjects where applicable.

The full detailed search strategies for all databases, including Boolean operators and syntax adaptations, are provided in Supplementary Table S1.

In addition, the reference lists of all included articles and relevant previous reviews were manually screened (snowballing technique) to identify additional eligible studies.

No additional limits on publication type or study design were applied during the initial search to ensure maximum sensitivity.

### 2.3. Eligibility Criteria

Studies were included if they were original research articles (retrospective or prospective cohort studies, registry-based analyses, or large administrative database studies) that met the following criteria: (1) evaluated adult patients (≥18 years) undergoing breast cancer surgery, including breast-conserving surgery, mastectomy, oncoplastic procedures, or any form of immediate or delayed reconstruction; (2) reported extractable postoperative outcomes within 30 days or during the index hospitalization; and (3) included data on at least one of the following domains: cardiovascular comorbidities (including heart failure), cardiovascular risk factors, or frailty assessment.

Clinically meaningful outcome measures were predefined as perioperative events with direct impact on patient safety, recovery, or healthcare utilization, including surgical complications, wound-related events, bleeding requiring intervention, cardiopulmonary complications, heart failure decompensation when reported, unplanned reoperation, hospital readmission, length of hospital stay, and 30-day mortality. Inclusion and exclusion criteria were prespecified to focus on original studies reporting 30-day or in-hospital postoperative outcomes in relation to cardiovascular comorbidity or frailty in adult breast cancer surgery patients, and to exclude reviews, case reports, and studies without extractable outcome data.

No minimum sample size threshold was imposed, provided that studies contributed clinically relevant outcome data.

Exclusion criteria were: (1) review articles, editorials, case reports, conference abstracts, or other forms of gray literature; (2) studies not focused on surgical treatment of breast cancer; (3) studies lacking extractable postoperative outcome data in relation to cardiovascular comorbidity or frailty; and (4) non-English publications.

Gray literature and conference abstracts were intentionally excluded due to limited methodological detail, lack of peer review, and potential risk of incomplete outcome reporting.

### 2.4. Study Selection

Study selection was conducted in two sequential stages. First, two independent reviewers (A.M. and M.M.) screened titles and abstracts to exclude clearly irrelevant records. Second, the same reviewers independently assessed full-text articles for eligibility according to the predefined inclusion and exclusion criteria.

Disagreements at any stage were resolved by discussion and consensus. Disagreements were resolved first by discussion between the two reviewers. If consensus could not be reached, a third senior reviewer (C.T. or D.A.S.) was consulted to reach the final decision.

**Figure 1:**
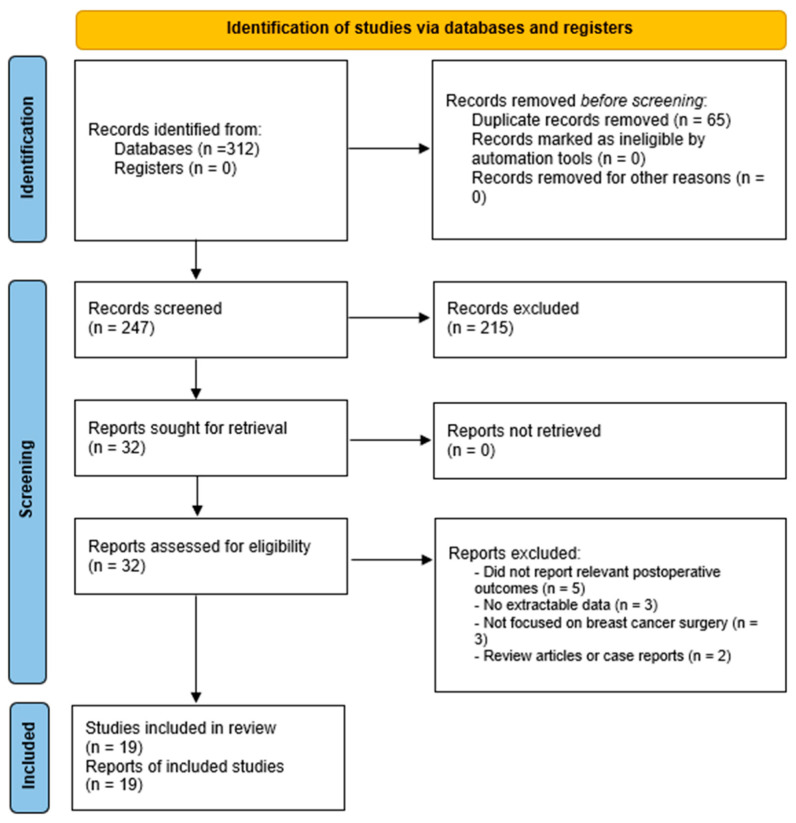
PRISMA 2020 flow diagram illustrating the study selection process, adapted for a systematized narrative review design. A total of 312 records were identified through database searching (PubMed/MEDLINE, Scopus, and Web of Science), with no additional records retrieved from registers. After removal of 65 duplicate records, 247 unique records were screened based on title and abstract, of which 215 were excluded. Full-text articles were sought and successfully retrieved for 32 studies, all of which were assessed for eligibility. Thirteen reports were excluded following full-text review due to lack of relevant postoperative outcomes (*n* = 5), insufficient or non-extractable data (*n* = 3), lack of focus on breast cancer surgery (*n* = 3), or ineligible publication type (review articles or case reports; *n* = 2). Ultimately, 19 studies were included in the qualitative synthesis [13,14,15,16,17,18,19,20,21,22,23,24,25,26,27,28,29,30,31].

Reasons for exclusion at the full-text stage are summarized in the PRISMA flow diagram (Figure 1), adapted for narrative review design.

### 2.5. Data Extraction and Synthesis

Data extraction was performed independently by two reviewers (D.-M.M. and I.C.) using a pre-piloted standardized Excel extraction form. The extracted variables included: study design and publication year; population characteristics (including age distribution and comorbidity profile); type and complexity of surgical intervention (breast-conserving surgery, mastectomy, reconstruction); presence and definition of cardiovascular comorbidities (with specific attention to heart failure when reported); frailty assessment method and score (e.g., modified frailty index, mFI-5); and all reported postoperative outcomes.

For each study, cardiovascular risk was characterized using the most specific data available, including explicit cardiovascular endpoints (e.g., cardiopulmonary complications, acute heart failure decompensation, cardiac events) when reported and, otherwise, broader systemic complications plausibly reflecting cardiovascular vulnerability (e.g., unplanned ICU admission, respiratory failure, sepsis, or composite medical complications). When cardiovascular outcomes were embedded in composite endpoints without separate reporting, this was recorded qualitatively and discussed as a limitation rather than quantitatively interpreted as a heart failure–specific signal.

Discrepancies between reviewers were resolved through discussion and consensus.

When data were missing, unclear, or incompletely reported in the original publications, this was documented qualitatively during the synthesis rather than imputed.

Given the heterogeneity of study designs, populations, and outcome definitions, findings were synthesized qualitatively. The results were organized into three prespecified domains: (1) surgical complexity; (2) cardiovascular vulnerability; and (3) frailty. This structured approach enabled an integrated interpretation of how procedural and patient-related factors interact to influence perioperative risk.

### 2.6. Conceptual Framework

To enhance clinical interpretability, the evidence was structured into a tri-axial conceptual framework integrating: (1) surgical technique complexity; (2) patient vulnerability, including cardiovascular comorbidities and frailty; and (3) postoperative outcomes.

This framework was used to guide both data synthesis and interpretation, allowing a clinically oriented analysis of how these domains interact to determine perioperative risk in breast cancer surgery.

**Figure 2:**
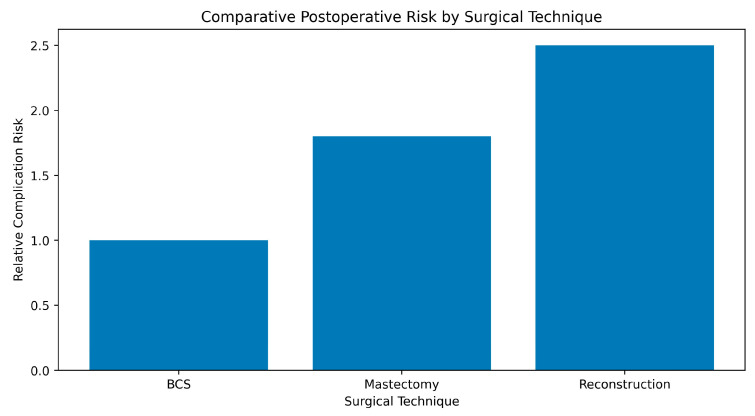
Schematic representation of the comparative postoperative complication risk according to surgical technique. Breast-conserving surgery (BCS) is generally associated with the lowest overall perioperative morbidity, whereas mastectomy and, particularly, reconstructive procedures are associated with progressively higher complication rates across the reviewed studies. The vertical axis represents relative complication risk on a qualitative scale, while the horizontal axis reflects increasing surgical complexity. This figure is schematic and summarizes qualitative trends from the included studies and should not be interpreted as a quantitative or meta-analyzed estimate.

**Figure 3:**
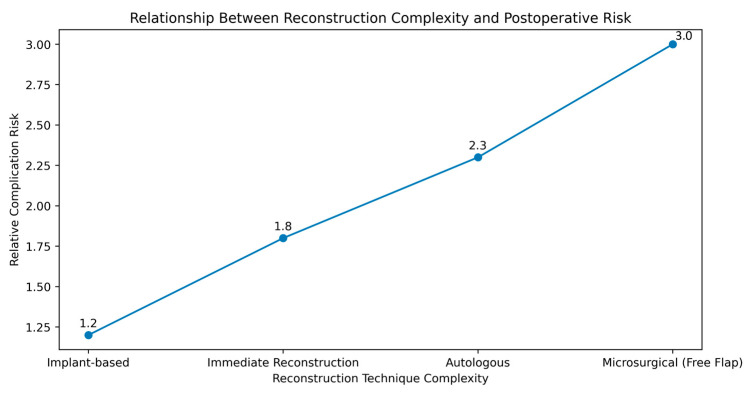
Schematic illustration of the relationship between reconstruction technique complexity and postoperative complication risk. Increasing procedural complexity—from implant-based to autologous and microsurgical free-flap reconstruction—is associated with progressively higher perioperative morbidity in the synthesized studies. The vertical axis represents relative complication risk on a qualitative scale, while the horizontal axis reflects increasing reconstruction complexity. This figure is schematic and summarizes qualitative trends from the included studies and should not be interpreted as a quantitative or meta-analyzed estimate.

**Figure 4:**
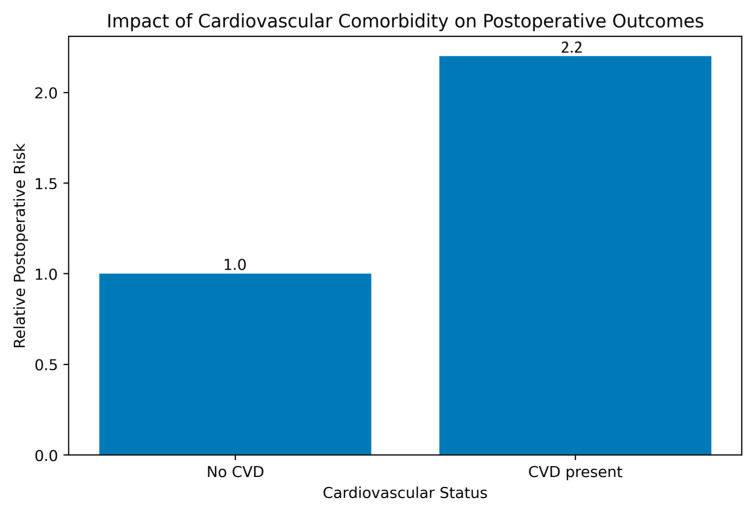
Schematic illustration of the impact of pre-existing cardiovascular comorbidity on postoperative outcomes. Patients with cardiovascular disease consistently demonstrate higher overall morbidity compared with those without, as observed across multiple studies. The vertical axis represents relative postoperative risk on a qualitative scale. This figure is schematic and summarizes qualitative trends from the included studies and should not be interpreted as a quantitative or pooled estimate.

**Figure 5:**
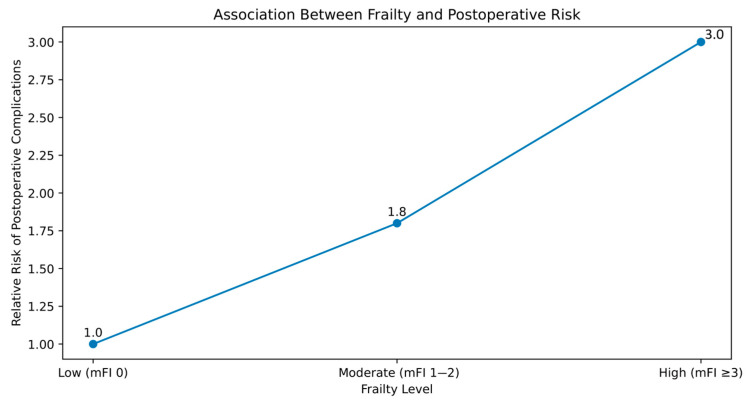
Schematic representation of the association between frailty (primarily assessed using the modified frailty index) and postoperative outcomes. Increasing frailty levels are associated with a stepwise rise in complications, mortality, and readmissions across breast surgery cohorts. The vertical axis represents relative risk on a qualitative scale. This figure is schematic and summarizes qualitative trends from the included studies and should not be interpreted as a quantitative or meta-analyzed estimate.

**Figure 6:**
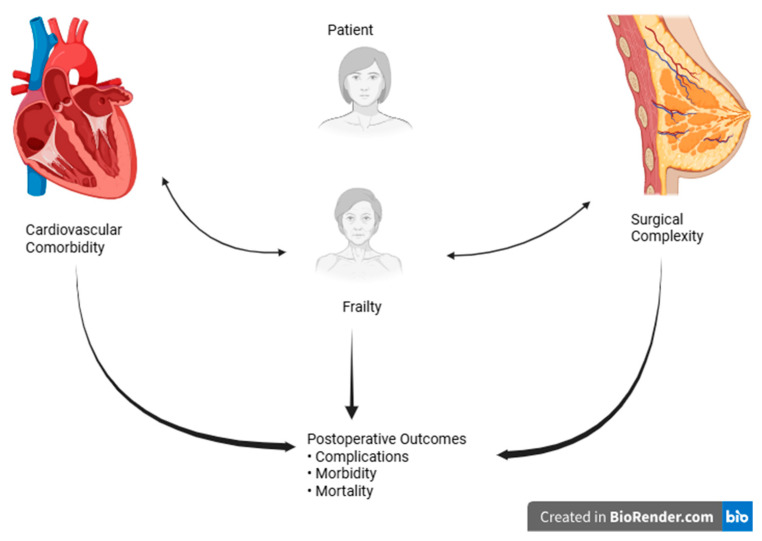
Conceptual integrated model of perioperative risk in breast cancer surgery. Surgical complexity, cardiovascular comorbidity (including heart failure vulnerability), and frailty interact dynamically to influence early postoperative outcomes. The model synthesizes findings from the 19 included studies into a clinically oriented framework. Arrows and relative risk levels are schematic/illustrative and do not represent statistically pooled hazard ratios or odds ratios.

The graphical representations included in this study (Figure 2, Figure 3, Figure 4, Figure 5 and Figure 6) are conceptual and illustrative in nature. They are derived from qualitative synthesis and are intended to facilitate clinical interpretation rather than to provide precise quantitative or data-driven estimates.

### 2.7. Risk of Bias and Limitations

Due to the narrative design and the high degree of heterogeneity among included studies (including differences in patient populations, surgical techniques, outcome definitions, and frailty assessment methods), a formal risk-of-bias assessment using standardized tools such as ROBINS-I was not performed.

Instead, a qualitative evaluation of methodological quality was undertaken. Preference was given to large-scale cohort studies, registry-based analyses, and studies derived from established databases such as ACS-NSQIP. Studies were critically appraised based on design (prospective vs. retrospective), sample size, adjustment for confounding variables, and clarity of outcome definitions.

Particular attention was paid to potential sources of bias, including selection bias, residual confounding, heterogeneity in cardiovascular phenotyping, and inconsistent or incomplete assessment of frailty. Additionally, the risk of misclassification of comorbidities and outcomes—especially in large administrative datasets—was considered.

A key limitation of the available evidence is the limited granularity and inconsistent reporting of cardiovascular and heart failure endpoints, with most studies focusing on composite postoperative complications rather than dedicated cardiovascular outcomes. Consequently, our synthesis primarily reflects overall perioperative vulnerability in patients with cardiovascular comorbidity and frailty, rather than precise estimates of heart failure event rates.

These methodological limitations, along with their implications for interpretation and generalizability, are further discussed in detail in the Section 4.

## 3. Results

### 3.1. Study Selection and General Characteristics

A total of 19 original studies [13,14,15,16,17,18,19,20,21,22,23,24,25,26,27,28,29,30,31] were included, comprising retrospective cohorts, population-based registry analyses, and large database studies (primarily ACS-NSQIP).

Several studies focused specifically on elderly populations and high-risk subgroups, demonstrating increased complication rates in these cohorts [14,15,16]. Additionally, large national datasets provided robust evidence regarding surgical outcomes and complication profiles across different operative strategies [17,18,19]. These studies evaluated diverse populations undergoing breast-conserving surgery (BCS), mastectomy, and reconstructive procedures. The main characteristics of the included studies are summarized in Table 1. The study selection process is illustrated in Figure 1.

### 3.2. Impact of Surgical Technique on Postoperative Outcomes

#### 3.2.1. Breast-Conserving Surgery Versus Mastectomy

Multiple studies consistently demonstrated that breast-conserving surgery is associated with lower perioperative morbidity compared to mastectomy, particularly in elderly patients [14,15]. In contrast, mastectomy may reflect postoperative complications, including both surgical and systemic events [16].

Large registry-based analyses confirmed that the extent of surgery is directly correlated with complication rates and healthcare burden [13,18].

Comparative postoperative risk according to surgical technique is illustrated in Figure 2.

#### 3.2.2. Oncoplastic Surgery

Oncoplastic breast-conserving techniques have been associated with favorable complication profiles. Comparative studies demonstrated lower surgical site complication rates compared to standard BCS [21].

However, more complex oncoplastic approaches may still increase perioperative risk in selected patients, particularly when associated with extensive resections or reconstruction [22].

### 3.3. Impact of Breast Reconstruction on Perioperative Risk

Reconstructive procedures were consistently associated with increased perioperative risk. Studies evaluating immediate breast reconstruction demonstrated higher rates of complications, including reoperation, bleeding, and wound-related events [19,20].

Risk-stratified analyses confirmed that reconstruction increases surgical morbidity, particularly in patients with comorbidities [18,23]. Furthermore, autologous and microsurgical reconstruction techniques were associated with increased operative complexity and complication rates compared to implant-based approaches [24,25].

Importantly, some studies demonstrated that reconstruction remains feasible in older patients, provided that careful selection criteria are applied [26].

The impact of different reconstruction techniques on postoperative outcomes is summarized in Table 2.

The relationship between reconstruction complexity and postoperative risk is illustrated in Figure 3.

### 3.4. Role of Cardiovascular Comorbidity in Surgical Outcomes

Cardiovascular comorbidity was identified as a significant predictor of postoperative complications. Patients with pre-existing cardiovascular disease exhibited higher rates of postoperative morbidity, including bleeding, reintervention, and systemic complications [13].

Large-scale analyses demonstrated that cardiovascular risk factors contribute significantly to adverse surgical outcomes, even in procedures considered low-risk [17].

Additionally, population-level data showed that postoperative bleeding risk may be influenced by systemic vascular pathology and comorbidity burden [18].

Although specific cardiovascular endpoints were variably reported, available evidence suggests that patients with underlying cardiac dysfunction, including heart failure, are at particularly increased risk of postoperative complications.

The reporting of specific cardiovascular outcomes across the included studies is summarized in Table 3. As shown, most studies captured cardiovascular vulnerability only within composite medical complications, with limited granularity regarding heart failure or discrete cardiac events. Importantly, detailed reporting of individual cardiovascular events-such as acute heart failure decompensation, myocardial infarction, or arrhythmias-was uncommon, with most datasets aggregating these outcomes into broad composite or “systemic complication” categories.

The impact of cardiovascular comorbidity on postoperative outcomes is illustrated in Figure 4.

### 3.5. Frailty as an Independent Predictor of Postoperative Complications

Frailty was one of the most consistent predictors of adverse outcomes. Studies using the modified frailty index demonstrated a stepwise increase in complication rates, mortality, and readmissions with increasing frailty scores [27,29]. Among the 19 included studies, frailty was formally assessed in three cohorts using the modified frailty index (mFI or mFI-5), whereas the remaining studies did not include a dedicated frailty instrument and instead reported age and comorbidity burden as proxies of vulnerability.

This association was observed across all surgical types, including both breast-conserving surgery and reconstructive procedures [30].

Recent studies further confirmed that frailty significantly impacts outcomes following mastectomy with and without reconstruction, emphasizing its role as a key determinant of perioperative risk [31].

The association between frailty and postoperative risk is illustrated in Figure 5.

### 3.6. High-Risk Subgroups

Elderly patients and those with metastatic disease were associated with significantly higher complication rates. Patients with metastatic breast cancer demonstrated increased postoperative morbidity, including cardiopulmonary complications [16].

Similarly, advanced age and comorbidity burden were consistently associated with worse outcomes across multiple studies [14,15].

### 3.7. Severe and Catastrophic Complications

Although rare, severe complications—including cardiovascular events and multi-organ failure—were reported in studies evaluating complex reconstructive procedures.

Microvascular free tissue transfer was associated with a measurable risk of catastrophic complications, including cardiovascular events and mortality [31].

### 3.8. Integrated Perspective

Across all studies, perioperative outcomes were associated with the interaction between: surgical complexity; cardiovascular comorbidity; frailty.

More invasive procedures (e.g., mastectomy with reconstruction) were associated with increased complication rates, particularly in frail or cardiovascularly compromised patients [18,27,31].

Conversely, less invasive approaches such as breast-conserving surgery were associated with improved safety profiles, especially in vulnerable populations [14,21].

The interplay between surgical complexity, cardiovascular comorbidity, and frailty, and their cumulative impact on postoperative outcomes, is summarized in Table 4.

An integrated conceptual model of perioperative risk, highlighting the interaction between surgical complexity, cardiovascular comorbidity, and frailty, is presented in Figure 6.

## 4. Discussion

### 4.1. Principal Findings in the Context of Existing Evidence

In this comprehensive narrative review, we synthesized evidence from 19 observational studies and large database analyses. Our aim was to delineate the interplay between surgical complexity, cardiovascular comorbidity, and frailty in shaping early postoperative outcomes after breast cancer surgery. We examined cohorts including elderly, metastatic, and reconstructive populations. Three consistent signals emerged. First, the extent of surgery—from breast-conserving surgery (BCS) to mastectomy and increasingly complex reconstruction—suggests perioperative complications and healthcare resource use [14,15,16,17,18,23]. Second, pre-existing cardiovascular disease and systemic vascular pathology were associated with increased postoperative risk, even in procedures traditionally considered low to intermediate cardiac risk [13,17,18]. Third, frailty, as captured by modified frailty indices, emerged as an independent and additive predictor of morbidity, mortality, and readmission, often outweighing chronological age [27,29,30]. Heart failure, in particular, represents a clinically critical yet underreported component of perioperative risk in breast cancer surgery. A key limitation of the current evidence base is the lack of granular reporting of cardiovascular events, with heart failure and other major cardiac outcomes rarely reported as discrete endpoints.

Importantly, given the observational nature of the included studies, these findings should be interpreted as associations rather than causal relationships. Selection bias is likely present, as frail or highly comorbid patients may have been preferentially directed toward less aggressive surgical strategies, and residual confounding cannot be excluded.

Our findings correspond with registry-based data showing that BCS is consistently associated with lower perioperative morbidity compared with mastectomy, particularly in older and comorbid women [14,15,16]. Studies using national datasets such as ACS-NSQIP confirm that escalation from BCS to mastectomy and immediate reconstruction is associated with stepwise increases in surgical and systemic complications, reoperation, and bleeding [18,20,23]. Reconstructive procedures—especially autologous and microsurgical free flap techniques—represent the highest-risk spectrum of breast surgery and are associated with measurable rates of severe and even catastrophic events in vulnerable patients [24,25,26,27,31]. Recent evidence suggests that carefully selected older patients may still benefit from reconstruction, provided that frailty and cardiovascular burden are explicitly integrated into perioperative decision-making [28].

In addition to primary studies, prior literature has explored surgical outcomes and reconstructive strategies in breast cancer, primarily focusing on complication profiles and procedural trends. Large-scale analyses have consistently reported increased complication rates associated with mastectomy and immediate reconstruction compared with breast-conserving approaches [32], while epidemiological studies have highlighted a progressive shift toward more complex surgical management over time [33]. More recent data further emphasize the evolving landscape of oncoplastic breast surgery, reflecting increasing technical complexity and individualized operative approaches [34].

Most existing studies and reviews have evaluated these factors in isolation, without integrating cardiovascular risk and frailty into a unified framework. Frailty has been widely recognized as a strong predictor of adverse surgical outcomes across multiple specialties [35]. Systematic evidence supports the role of comprehensive geriatric assessment in improving perioperative risk stratification [36]. However, the interaction between surgical complexity, cardiovascular comorbidity, and frailty remains insufficiently explored in the specific context of breast cancer surgery. By synthesizing evidence across these domains, our review provides an integrated and clinically applicable model of perioperative risk, while acknowledging the inherent limitations of the available observational data. To our knowledge, this is the first review to integrate surgical complexity, cardiovascular vulnerability, and frailty into a unified, clinically applicable perioperative risk framework in breast cancer surgery, and as such it addresses an important gap in the existing literature

### 4.2. Surgical Technique, Cardiovascular Risk, and Frailty: Mechanistic and Clinical Implications

The consistent gradient of risk from BCS to mastectomy and then to reconstruction likely reflects both procedural and patient-selection mechanisms. More extensive operations are associated with longer anesthesia duration, greater blood loss, larger wound surfaces, and more pronounced inflammatory and hemodynamic stress [37,38]. These factors may unmask subclinical cardiovascular instability [18,23,25]. Large nationwide and NSQIP analyses show that postoperative bleeding and systemic complications increase with surgical complexity [18,23]. This is consistent with the hypothesis that the surgical insult interacts with underlying vascular and cardiac vulnerability. In this context, pre-existing heart failure, ischemic heart disease, and vascular pathology may lower the threshold for perioperative decompensation [13,17].

Frailty adds a distinct, yet overlapping, dimension to cardiovascular risk. Modified frailty indices capture deficits across functional, cognitive, and comorbid domains. These indices reflect a diminished physiological reserve and impaired ability to respond to surgical stress. Studies applying the modified frailty index reported a stepwise increase in complications, mortality, and 30-day readmissions with rising frailty scores, independent of age [27,29,30]. The impact of frailty remained evident across the spectrum from BCS to microsurgical reconstruction. Even procedures with relatively low absolute risk may become high-risk when performed in frail patients [27,30].

Oncoplastic BCS occupies a nuanced position within this continuum. These techniques can achieve favorable complication profiles and improved aesthetic outcomes [21,22]. Their increasing technical complexity demonstrates a broader shift toward individualized surgical strategies [34]. However, this evolution must be balanced against the potential for increased physiological stress, particularly in vulnerable patient populations.

For example, in an elderly patient with preserved functional status but significant heart failure (NYHA II–III) and high mFI score, prioritizing breast-conserving surgery or delaying complex reconstruction may reduce perioperative risk. Conversely, in a younger, non-frail patient with controlled cardiovascular risk factors, immediate reconstruction-including more complex techniques-may be considered within a multidisciplinary setting. This framework is conceptually aligned with existing ESC cardio-oncology guidelines and perioperative risk stratification models, which emphasize the importance of cardiovascular evaluation, functional status, and global patient risk in surgical decision-making. However, our model extends these principles by explicitly integrating frailty and surgical complexity into a unified, clinically oriented perspective.

### 4.3. High-Risk Subgroups and Opportunities for Personalized Surgical Planning

Elderly and metastatic patients consistently emerged as high-risk subgroups across the included studies. Population-based analyses in older women show higher rates of both surgical and medical complications following mastectomy compared with BCS [14,15,16]. In metastatic disease, national database data reveal increased postoperative morbidity, including cardiopulmonary events, when extensive surgery or reconstruction is pursued [17].

Complex reconstruction, particularly microsurgical free tissue transfer, represents another critical high-risk domain. Large NSQIP-based analyses document not only higher overall complication and reoperation rates, but also a non-trivial incidence of catastrophic events [25,31]. While these procedures can offer superior aesthetic and psychosocial outcomes, our review suggests they should be reserved for patients with adequate cardiovascular reserve and low frailty [26,31].

An important insight from the included frailty studies is that chronological age alone is an insufficient surrogate for vulnerability. Older but non-frail individuals can experience acceptable complication rates after reconstruction, whereas younger yet frail patients may face disproportionate risk [27,29,30].

Based on the proposed framework, a simplified risk-adapted approach may include: (1) Assess surgical complexity (BCS vs. mastectomy vs. reconstruction); (2) Evaluate cardiovascular status, including heart failure and functional capacity; (3) Assess frailty (e.g., mFI where available); (4) Integrate these domains to guide surgical strategy, favoring less invasive approaches or staged reconstruction in high-risk patients.

To enhance clinical applicability, we propose a structured clinical decision algorithm based on the tri-axial framework. This proposed algorithm is intended as a pragmatic clinical tool to support individualized surgical decision-making rather than to replace guideline-based risk assessment. It is intended to support clinical reasoning rather than replace individualized guideline-based assessment.

Step 1: Define surgical complexity:

- Low: Breast-conserving surgery (BCS) ± oncoplastic techniques
- Intermediate: Mastectomy (simple or skin-sparing)
- High: Mastectomy with immediate reconstruction (implant-based, autologous, or microsurgical free flap)

Step 2: Evaluate cardiovascular vulnerability (preoperative cardio-oncologic assessment)

- History of heart failure (NYHA class)
- Other major CV comorbidities (ischemic heart disease, valvular disease, arrhythmias)
- Functional capacity (METs or Duke Activity Status Index–DASI)
- Consider echocardiography, BNP/NT-proBNP if heart failure suspected or high-risk features present

Step 3: Assess frailty

- Use validated tool: mFI-5 (preferred in NSQIP-compatible settings) or equivalent
- If available and patient ≥65 years or perceived frail: Comprehensive Geriatric Assessment (CGA)
- mFI-5 score interpretation: 0–1 = non-frail; ≥2 = frail (higher scores indicate greater vulnerability)

Step 4: Integrated risk stratification and procedure selection

- Low risk (non-frail + no significant CVD): Full range of options possible, including immediate complex reconstruction when oncologically and aesthetically indicated.
- Intermediate risk (moderate frailty or controlled CVD): Limit complexity–prefer BCS or mastectomy without immediate reconstruction; consider staged/delayed reconstruction.
- High risk (frail [mFI-5 ≥ 2–3] + significant CVD or heart failure): Strongly favor de-escalated approach (BCS when feasible or mastectomy alone); avoid immediate autologous/microsurgical reconstruction; prioritize symptom control and quality of life.

Step 5: Multidisciplinary team (MDT) discussion and shared decision-making

- Involve breast surgeon, plastic surgeon, cardiologist/cardio-oncologist, geriatrician (if frail), and patient.
- Optimize modifiable factors (heart failure therapy, prehabilitation, anemia correction) before proceeding.
- Document rationale and align with patient values and oncologic priorities.

This algorithm integrates the tri-axial framework (surgical complexity × cardiovascular vulnerability × frailty) and is aligned with contemporary perioperative cardiovascular recommendations (including AHA/ACC and ESC guidance, such as the 2022 ESC Cardio-Oncology Guidelines). It must be emphasized that this proposed algorithm is hypothesis-generating and has not been prospectively validated in dedicated clinical cohorts. It is intended to support structured clinical reasoning and facilitate multidisciplinary discussion rather than to replace individualized, guideline-based perioperative assessment.

### 4.4. Strengths, Limitations, and Future Directions

Although the title and conceptual framework emphasize heart failure as a key determinant of perioperative risk, the primary data synthesized in this review predominantly reflect broader cardiovascular comorbidity, with only limited HF-specific event reporting in the underlying studies.

This review has several notable strengths. We conducted a broad, multi-database search (PubMed/MEDLINE, Scopus, Web of Science) up to 31 January 2026. Our focus was on original research that explicitly reported postoperative outcomes in relation to comorbidities, cardiovascular risk factors, or frailty among patients undergoing BCS, mastectomy, and reconstruction. Preference was given to large cohort studies, national registries, and NSQIP-based analyses. This approach enhanced the external validity and contemporary relevance of our synthesis. By structuring the evidence into domains of surgical complexity, cardiovascular vulnerability, and frailty, we propose an integrated, clinically applicable conceptual framework rather than a purely descriptive aggregation of complication rates.

However, important limitations must be acknowledged. The structured narrative design allows integrative interpretation across heterogeneous studies but limits quantitative synthesis and may introduce subjective elements in data interpretation. Importantly, given the observational nature of the included studies, the reported relationships should be interpreted as associations rather than causal effects. Most included studies were retrospective and observational, making them susceptible to selection bias, residual confounding, and misclassification of comorbidities and postoperative events. In particular, selection bias related to surgical decision-making is likely present, as frail or highly comorbid patients may have been preferentially directed toward less invasive procedures.

Cardiovascular outcomes were frequently embedded within broad composite endpoints (e.g., “medical” or “systemic” complications), with limited granularity regarding specific event types, such as acute heart failure decompensation, myocardial infarction, or arrhythmias, and limited detail regarding event timing and mechanisms. Frailty was assessed using different versions of the modified frailty index, and many large datasets lacked frailty measures altogether. A further limitation is that frailty was captured almost exclusively using modified frailty indices (mFI or mFI-5) derived from administrative or NSQIP variables, rather than through multidimensional clinical instruments. While mFI-based scores have been validated as pragmatic predictors of postoperative risk, they primarily reflect comorbidity and functional deficits and may underestimate cognitive, social, and nutritional dimensions of vulnerability. As a result, our findings likely represent a conservative estimate of the true impact of frailty on perioperative outcomes.

None of the included datasets incorporated a formal comprehensive geriatric assessment (CGA), despite robust evidence that CGA improves perioperative risk stratification in older surgical patients. This gap may partly explain why frailty is underreported and reinforces the need for prospective breast surgery cohorts integrating standardized geriatric assessment into preoperative workflows. Most data come from high-income healthcare systems, which may limit generalizability to settings with different resources, perioperative pathways, and case mix.

These gaps define a clear agenda for future research. Prospective, multicenter cohorts incorporating standardized frailty instruments, detailed cardiovascular phenotyping, and procedure-specific complication definitions are needed to refine risk prediction in breast cancer surgery. Pragmatic randomized or well-designed observational studies comparing de-escalated surgical strategies (e.g., BCS or omitting immediate reconstruction) against more complex procedures in frail or cardiovascularly compromised patients would help quantify trade-offs between oncologic control, functional outcomes, and perioperative safety. Additionally, interventional trials of prehabilitation and targeted cardiovascular optimization in high-risk breast surgery candidates could clarify whether modifiable factors may reduce postoperative morbidity. Finally, integrating the proposed tri-axial framework of surgical complexity, cardiovascular comorbidity, and frailty into decision aids and guideline algorithms may support more individualized, patient-centered care in an aging breast cancer population.

## 5. Conclusions

This narrative review shows that early postoperative outcomes after breast cancer surgery are associated with the dynamic interaction between surgical complexity, cardiovascular vulnerability, and frailty, rather than by operative technique in isolation. Across contemporary cohorts and large databases, escalation from breast-conserving surgery to mastectomy and increasingly complex reconstruction was accompanied by a graded rise in perioperative morbidity, with the highest risk observed in elderly, frail, and comorbid patients. Cardiovascular disease and systemic vascular pathology consistently amplified this gradient, while frailty—captured by modified frailty indices—emerged as a powerful, age-independent marker of adverse events, including complications, mortality, and readmission.

These observations support a decisively patient-centered, risk-adapted approach to breast cancer surgery, in which operative strategies are deliberately aligned with each patient’s cardiovascular profile and frailty status. In vulnerable individuals, privileging less invasive procedures, reconsidering the timing and complexity of reconstruction, and embedding standardized frailty and cardio-oncologic assessment into preoperative workflows may substantially improve safety without undermining oncologic intent. Conversely, in robust patients with preserved physiological reserve, even advanced reconstructive options can be undertaken safely within multidisciplinary pathways and experienced centers. Future prospective and interventional studies should validate this integrated framework and test structured optimization and prehabilitation strategies, with the goal of translating nuanced risk stratification into tangible reductions in postoperative medical complications in an aging breast cancer population. Particular attention should be given to patients with underlying heart failure, in whom perioperative risk may be substantially amplified even in procedures traditionally considered low-risk.

## Figures and Tables

**Table 1:** Characteristics of included studies evaluating surgical technique, cardiovascular comorbidity, and frailty in breast cancer surgery (*n* = 19).

Study	Year	Study Design	Population	Surgical Technique	Cardiovascular Data	Frailty Assessment	Main Outcomes
Huang et al. [13]	2016	Cohort	Breast cancer patients	Mixed	Yes	No	Postoperative complications
de Boniface et al. [14]	2023	Registry-based	Elderly women	BCS vs. mastectomy	Partial	No	Surgical & medical complications
Pettke et al. [15]	2016	Cohort	Elderly patients	Mixed	Partial	No	Short-term outcomes
Rocco et al. [16]	2013	Cohort	Elderly patients	Mixed	Yes	No	Complications & survival
Cordeiro et al. [17]	2014	Database (NSQIP)	Metastatic BC	Mixed	Partial	No	Postoperative morbidity
Konishi et al. [18]	2022	Nationwide database	Breast surgery patients	Mixed	Yes	No	Bleeding risk
Saha et al. [19]	2013	Cohort	Reconstruction patients	Reconstruction	Yes	No	Complications
Al-Hilli et al. [20]	2015	NSQIP	Breast surgery patients	BCS vs. mastectomy	Partial	No	Reoperation
Crown et al. [21]	2019	Cohort	BCS patients	Oncoplastic vs. standard	No	No	Surgical complications
Oberhauser et al. [22]	2021	Cohort	BCS patients	Oncoplastic	Partial	No	Complications & recurrence
Jonczyk et al. [23]	2019	NSQIP (13-year)	Breast surgery patients	Mixed	Yes	No	Acute complications
Saheb-Al-Zamani et al. [24]	2021	NSQIP	Reconstruction patients	Reconstruction	Partial	No	Early complications
Fischer et al. [25]	2013	NSQIP	Reconstruction patients	Immediate reconstruction	Yes	No	Surgical morbidity
Gart et al. [26]	2013	NSQIP	Reconstruction patients	Autologous vs. implant	Partial	No	Complications
Ali et al. [27]	2022	Cohort	Reconstruction patients	Microsurgical	Yes	mFI (11-item NSQIP modified frailty index)	30-day complications
Dolen et al. [28]	2022	Cohort	Elderly patients	Reconstruction	Partial	No	Feasibility & outcomes
Knoedler et al. [29]	2025	Cohort	Breast cancer patients	BCS	No	mFI-5 (5-item modified frailty index)	Complications
Bilgili et al. [30]	2025	Cohort	Breast surgery patients	Mastectomy ± reconstruction	Yes	mFI-5 (5-item modified frailty index)	Morbidity
Diaddigo et al. [31]	2024	NSQIP	Reconstruction patients	Microsurgical	Yes	No	Catastrophic complications

Cardiovascular Data: “Yes” indicates that dedicated cardiovascular comorbidities or risk factors were explicitly analyzed as predictors; “Partial” indicates that cardiovascular variables were reported but only as part of broader comorbidity indices or composite endpoints, without specific or independent analysis. Frailty Assessment: “mFI” or “mFI-5” indicates the use of a modified frailty index; “No” indicates that frailty was not formally assessed, with age and/or comorbidity burden used as proxy measures.

**Table 2:** Impact of Breast Reconstruction Techniques on Postoperative Outcomes.

Study	Reconstruction Type	Comparison	Complications	Reoperation	Key Findings
Saha et al. [19]	Immediate reconstruction	Risk-stratified analysis	↑ Increased	Yes	Reconstruction associated with higher complication rates
Saheb-Al-Zamani et al. [24]	Immediate reconstruction	Timing & technique	↑ Increased	Yes	Early postoperative complications influenced by technique
Fischer et al. [25]	Immediate reconstruction	Risk stratification	↑ Increased	Yes	Higher surgical morbidity in complex cases
Gart et al. [26]	Autologous vs. implant	Comparative	↑↑ Autologous > Implant	Yes	Autologous reconstruction associated with higher morbidity
Ali et al. [27]	Microsurgical reconstruction	Frailty-based analysis	↑ Increased	Yes	Frailty strongly predicts complications (mFI)
Dolen et al. [28]	Reconstruction in elderly	Feasibility	Moderate	No	Reconstruction feasible in selected elderly patients
Bilgili et al. [30]	Mastectomy ± reconstruction	Comparative	↑ Increased	Yes	Reconstruction increases perioperative risk
Diaddigo et al. [31]	Microsurgical free flap	Complex procedures	↑↑ High	Yes	Risk of catastrophic complications

↑ indicates increased risk; ↑↑ indicates markedly increased/high risk. These symbols represent qualitative trends derived from the included studies and do not correspond to quantitative effect sizes.

**Table 3:** Reporting of cardiovascular and cardiopulmonary outcomes in the included studies (*n* = 19).

Study	Population/ Surgery Type	Cardiovascular/Cardiopulmonary Endpoints Reported	Reporting Format	Key Cardiovascular-Related Findings
Huang et al. [13]	Mixed breast cancer surgery	Preoperative CVD risk factors; general postoperative complications	Composite/risk factors only	Preoperative CVD burden associated with higher overall morbidity
de Boniface et al. [14]	Elderly women (BCS vs. mastectomy)	Medical complications (including cardiopulmonary)	Broad medical complications	Higher medical complications after mastectomy in elderly
Pettke et al. [15]	Elderly patients, mixed	Short-term outcomes (unspecified cardiopulmonary)	General complications	Increased complications with advanced age
Rocco et al. [16]	Elderly patients, mixed	Complications & survival (cardiopulmonary mentioned)	Composite	Higher postoperative complications in elderly
Cordeiro et al. [17]	Metastatic breast cancer, mixed	Postoperative morbidity (including cardiopulmonary)	Composite morbidity	Higher overall morbidity in metastatic disease
Konishi et al. [18]	Nationwide breast surgery	Postoperative bleeding (vascular-related)	Specific (bleeding)	Systemic vascular pathology increased bleeding risk
Saha et al. [19]	Reconstruction patients	General complications (cardiac not separately reported)	Composite	Reconstruction associated with higher complications
Al-Hilli et al. [20]	BCS vs. mastectomy (NSQIP)	Reoperation and complications (cardiac embedded)	Composite	Higher reoperation rates with more extensive surgery
Crown et al. [21]	Oncoplastic vs. standard BCS	Surgical site complications only	Surgical only	No specific cardiovascular reporting
Oberhauser et al. [22]	Oncoplastic BCS	Complications & recurrence (cardiac not detailed)	General complications	Favorable profile but limited CV data
Jonczyk et al. [23]	Mixed breast surgery (13-year NSQIP)	Acute complications (including cardiopulmonary)	Composite acute complications	Trends toward safer surgeries over time
Saheb-Al-Zamani et al. [24]	Reconstruction (timing & technique)	Early postoperative complications	Composite	Technique influences early complications
Fischer et al. [25]	Immediate reconstruction (NSQIP)	Surgical morbidity (cardiac risk factors noted)	Composite + risk stratification	Higher morbidity in complex cases
Gart et al. [26]	Autologous vs. implant reconstruction	Complications (cardiac not separated)	Comparative complications	Autologous higher morbidity
Ali et al. [27]	Microsurgical reconstruction	30-day complications (frailty + CV)	mFI + complications	Frailty strongly predicted complications
Dolen et al. [28]	Reconstruction in elderly	Feasibility & outcomes (medical complications)	General outcomes	Feasible in selected elderly
Knoedler et al. [29]	BCS	Complications using mFI-5	Frailty-focused	mFI-5 predicted complications
Bilgili et al. [30]	Mastectomy ± reconstruction	Morbidity (CV and frailty)	Frailty + morbidity	Frailty associated with higher morbidity
Diaddigo et al. [31]	Microsurgical free flap	Catastrophic complications (including cardiovascular events)	Specific catastrophic events	Measurable risk of cardiovascular events and mortality in complex reconstruction

CVD = cardiovascular disease; BCS = breast-conserving surgery; NSQIP = National Surgical Quality Improvement Program; mFI = modified frailty index. Most studies reported cardiovascular outcomes only as part of broad composite endpoints (e.g., “medical complications” or “systemic events”) rather than as discrete heart failure or major adverse cardiac events. Dedicated cardiovascular endpoints (e.g., acute heart failure decompensation, myocardial infarction, arrhythmia) were rarely reported separately. This limited granularity restricts precise attribution of postoperative risk to specific cardiovascular events, including heart failure.

**Table 4:** Integrated Determinants of Postoperative Risk in Breast Cancer Surgery.

Domain	Factor	Evidence from Studies	Impact on Outcomes	Clinical Implications
Surgical Complexity	Breast-conserving surgery (BCS)	[14,21]	↓ Lower complication rates	Preferred in high-risk patients
	Mastectomy	[14,15,16,18]	↑ Increased morbidity	Careful patient selection required
	Reconstruction (overall)	[19,20,23,24,25,26]	↑ Increased complications	Risk–benefit assessment necessary
	Autologous/microsurgical reconstruction	[24,25,26,27,31]	↑↑ Highest complication risk	Reserve for selected patients
Cardiovascular Comorbidity	Pre-existing CVD	[13,17,18]	↑ Increased postoperative complications	Preoperative optimization essential
	Systemic vascular pathology	[18]	↑ Bleeding and systemic risk	Multidisciplinary management
Frailty	Increased frailty index (mFI)	[27,29,30]	↑↑ Strong predictor of morbidity and mortality	Routine frailty assessment recommended
High-Risk Subgroups	Elderly patients	[14,15,16,28]	↑ Increased complication rates	Individualized surgical planning
	Metastatic disease	[17]	↑ Higher postoperative morbidity	Conservative strategies preferred

↓ indicates reduced risk; ↑ indicates increased risk; ↑↑ indicates markedly increased risk. These symbols reflect qualitative trends based on the included studies and should not be interpreted as quantitative or meta-analytic estimates.

## Data Availability

All data analyzed in this study were obtained from previously published articles cited in the reference list. The extracted dataset and statistical analysis files are available from the corresponding author upon reasonable request.

## Supplementary Materials

The following supporting information can be downloaded at: https://www.mdpi.com/article/10.3390/medicina62050877/s1, Table S1: Detailed search strategies used in the literature search.

## Abbreviations

The following abbreviations are used in this manuscript:

ACS-NSQIP	American College of Surgeons National Surgical Quality Improvement Program
BCS	Breast-Conserving Surgery
CI	Confidence Interval
CVD	Cardiovascular Disease
ESC	European Society of Cardiology
HER2	Human Epidermal Growth Factor Receptor 2
IC-OS	International Cardio-Oncology Society
MeSH	Medical Subject Headings
mFI	Modified Frailty Index
mFI-5	5-Item Modified Frailty Index
NSQIP	National Surgical Quality Improvement Program
PRISMA	Preferred Reporting Items for Systematic Reviews and Meta-Analyses
