# Supplementary material for: Cardiovascular Vulnerability, Including Heart Failure Risk, in Breast Cancer Surgery: The Role of Operative Technique, Frailty, and Postoperative Complications
Source: Medicina (Kaunas). 2026 May 3;62(5):877. doi: 10.3390/medicina62050877 (PMC13208202; doi:10.3390/medicina62050877)
Supplement: Supplementary file 1 [file medicina-62-00877-s001.zip › medicina-4236369-supplementary.pdf]

Supplementary Materials

Supplementary Table S1. Detailed search strategies used in the literature search.

Database	Full Search Strategy (with field codes)	Limits / Filters Applied	Date of Last Search
PubMed/MEDLINE	("breast cancer surgery"[Title/Abstract] OR mastectomy[Title/Abstract] OR "breast-conserving surgery"[Title/Abstract] OR "oncoplastic surgery"[Title/Abstract] OR "breast reconstruction"[Title/Abstract] OR "mastectomy"[MeSH Terms] OR "breast neoplasms/surgery"[MeSH Terms]) AND ("cardiovascular disease"[Title/Abstract] OR "heart failure"[Title/Abstract] OR "cardiac risk"[Title/Abstract] OR "perioperative risk"[Title/Abstract] OR "postoperative complications"[Title/Abstract] OR frailty[Title/Abstract] OR "frailty index"[Title/Abstract] OR "modified frailty index"[Title/Abstract] OR "heart failure"[MeSH Terms] OR "cardiovascular diseases"[MeSH Terms])	English language, Humans	31 January 2026
Scopus	TITLE-ABS-KEY(("breast cancer surgery" OR mastectomy OR "breast-conserving surgery" OR "oncoplastic surgery" OR "breast reconstruction") AND ("cardiovascular disease" OR "heart failure" OR "cardiac risk" OR "perioperative risk" OR "postoperative complications" OR frailty OR "frailty index" OR "modified frailty index"))	English language	31 January 2026
Web of Science	TS= (("breast cancer surgery" OR mastectomy OR "breast-conserving surgery" OR "oncoplastic surgery" OR "breast reconstruction") AND ("cardiovascular disease" OR "heart failure" OR "cardiac risk" OR "perioperative risk" OR "postoperative complications" OR frailty OR "frailty index" OR "modified frailty index"))	English language	31 January 2026
